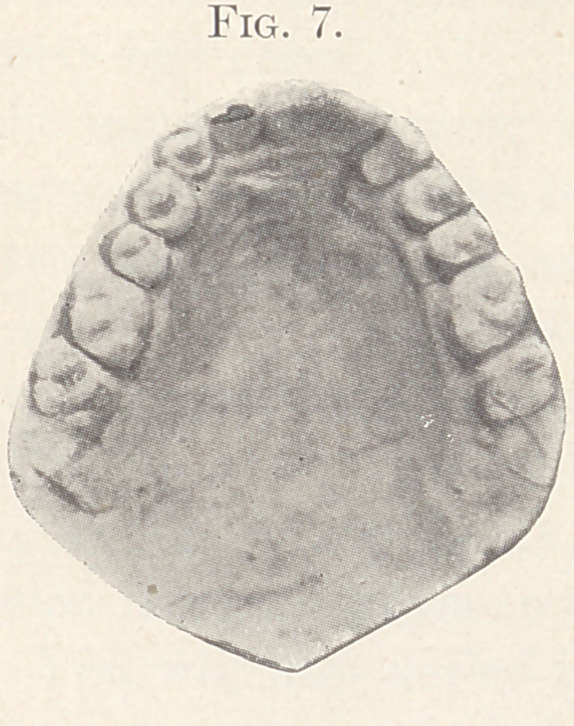# Physiological Results of Operation for Cleft Palate

**Published:** 1902-08

**Authors:** Thomas Fillebrown

**Affiliations:** Boston, Mass.


### PHYSIOLOGICAL RESULTS OF OPERATION FOR CLEFT PALATE.[1]

BY DR. THOMAS FILLEBROWN, BOSTON, MASS.

My paper to-night is very brief, for the reason that this matter has been discussed here sufficiently for every member of the Academy to understand what I do, how I do it, and what I do it for. All I have to present now are the results. I have four patients present. I hope to have another, but the difficulty of getting patients of this description together for any one date cannot be fully realized until it is tried. I fully expected to have here a young boy on whom I operated at six years of age, the pictures of whose mouth and lip you have seen here, and which were published in the International Dental Journal of September, 1898. His family have lately moved away, and that has made it impossible for him to be here. His is one of the most remarkable —I should say the most remarkable case I have ever had, because there was so much to overcome,—an absolutely disabling deformity

---

[1] Read before the American Academy of Dental Science, February 5, 1902.

of the lip with an exceedingly large cleft in the mouth. The boy is talking plainly to-day. The patient's upper lip, while long enough, was so narrow that it was absolutely immovable by any muscular power he possessed. The operation on the lip consisted of widening the lip by taking a portion of the cheek at each corner of the mouth and transferring it to the lip, making the lip broad enough so that he could project it in speaking and raise it so as to show his teeth, and command it to whistle.

The lips have much to do with pronunciation. The patient who has simply a cleft of the palate to be closed, and uses his lips with any degree of skill, can talk without difficulty, but the person who has a cicatrix in the lip and cannot use it at all has much greater difficulty to overcome. But even those patients who have large cicatrices, if their lips are put into proper form and are properly educated in use, are able to overcome that difficulty to a degree that is surprising to one as great an optimist as myself.

Some of these patients were operated on at the clinic of the Harvard Dental School and some of them are private patients, but I wish to say that whether the patients are private or clinic, this work is all the outcome of the surgical clinic of the Harvard Dental School, and but for that clinic I do not believe I would have the pleasure of presenting them here this evening. The credit of it all belongs to that institution.

Fig. 1 shows the mouth of a patient I will first present as it was before the operation. I have no model of it since the operation of the palate as complete. The patient is wearing an artificial set of teeth held by atmospheric pressure. The teeth were so elongated and out of position that it seemed unwise to attempt to preserve them. This lady was forty years old when I operated on her mouth and closed the cleft. Previous to that her enunciation was extremely indistinct, so that she could be understood only by persons knowing what she meant by the sounds she made. She is a dressmaker and a little hard of hearing, and of course makes no pretence to skill as a public speaker. The especial point I wish to be appreciated is the advanced age of the lady when her mouth was operated on, and notice that to-night she is speaking so plainly as to be perfectly well understood, when before it was impossible for a stranger to understand her.

(The patient appears and repeats the Lord's Prayer and answers questions.)

Fig. 1.

Fig. 2.

Fig. 3.

Fig 4.

Fig. 5.

Fig. 6.

Fig. 7.

*Dr. Werner.*—How long ago was this operated on?

*Dr. Fillebrown.*—A year ago last August. I subsequently took out the teeth because I could not obtain any reasonable result with them in.

*Dr. Werner.*—Can she talk as well when she has not her plate in?

*Dr. Fillebrown.*—She shows only the difference that any person does who wears a plate.

Figs. 2 and 3 show the mouth of a child nine years old before and after operation. The operation was done two years ago. Within three or four days after the operation, when the swelling had begun to subside, I began to talk to her, and I found that under my direction she could speak about as plainly as she does now. She will tell the society something about where she lives, etc. (She gives her address, repeats poetry, and answers numerous questions with normal distinctness.)

*Dr. Brackett.*—Will you state the condition of the patient previous to the operation?

*Dr. Fillebrown.*—Fig. 2 shows it very nicely. The cleft included the soft palate and extended a little into the hard palate.

*Dr. Meriam.*—What was the age of the patient at the time.

*Dr. Fillebrown.*—She was nine years old.

(Patient speaks up: "I am eleven years old now.")

*Dr. Fillebrown.*—You see how active her lips are when articulating; that accounts for the perfection of her speech. I think you must feel there is no question about her speaking so as to be understood.

I have two cases here which were operated on quite recently. They are not perfect in speech. I do not present either one of them as such. I present them to show you the immediate results that are gained in extremely bad cases.

Figs. 4 and 5 show the mouth of a young lady eighteen years old. I operated on her palate last July. The lip presented a deep notch, and the left nostril was very broad and lay almost flat against her face. Last August I corrected this deformity. This photograph of the patient shows what the lip was before the operation, and now we present the lip itself. This case was not finished until the 26th of August, so that it is only six months old. You will notice the cleft extended from the uvula to the lateral incisors. It is all closed up now. You will also notice in this, as in every other case, that *s* is the only hard letter to manage. Almost all the patients get over the trouble by minimizing the sound of *s* by a sort of skipping over it. This patient has not yet learned how to do this, and I doubt if she will ever need to, for I think she will entirely conquer the sound after a little. The soft palate is flexible and growing more and more so every day.

(Patient repeats different couplets, among them, "Theophilus Thistle the successful thistle sifter," etc.)

Fig. 6 represents the mouth of a young man twenty-three years old before operation. Fig. 7 shows the same mouth as it is at present. The cleft you will notice is very wide and the arch of the mouth is high. This latter circumstance is favorable to the operation. This patient previous to my operation called on experts, both dental and medical, and was told by all, so he says, that no man living could close the cleft surgically. I wish only to say that I present the patient with the cleft closed, and the man that did it still lives. The nose and lip were in very much the same condition as those of my last patient. I operated on the lip and nose the same as in the previous case. I made two operations for the closure of the cleft, the first on the hard palate, the second on the soft palate some weeks later. There is still an opening about one-thirty-second of an inch wide by one-eighth of an inch at the junction of the two palates to be closed up. (This opening has subsequently been closed by three applications of strong carbolic acid.) His speech was more indistinct than that of these other patients. The patient has worn a small obturator to cover this opening, so that his speech has progressed the same as if it was entirely closed up. The surgical operations were completed last summer.

(To the patient.) When did you have the first operation?

*Patient.*—I had the first operation on the mouth the 19th of last February, nearly a year ago. I had the last operation the 11th day of last June, and I am yet to have one more, which I think will prove successful, and I expect to be far better than I am now.

*Dr. Fillebrown.*—If you choose to take the time, he would be very glad to have you look at his mouth. He talked with me over the telephone the other day, and I did not realize there was any trouble with him at all. In the course of the talk there was just one word I could not understand, and that was congress, and I

answered back what I thought it was, and he did not understand me, so we were even.

*Dr. Smith.*—I would like to ask Dr. Fillebrown if the young man wore an obturator before the operation?

*Patient.*—No, sir, I did not.

*Dr. Fillebrown.*—You will see in these patients the effort that is made to use the lip. By that means the good articulation is obtained; without that it would be difficult to understand them.

*A member.*—Is your speech a good deal improved now over what it was two years ago?

*Patient.*—Yes, wonderfully.

*A member.*—Was it hard to make people understand you then?

*Patient.*—Yes, very hard. I find a large improvement already. I expect more as time goes on. I have no trouble, hardly, now in making them understand me.

*Dr. Baker.*—I would like to ask Dr. Fillebrown what vocal instructions the patient has had?

*Dr. Fillebrown.*—I have given him what he has had.

*Dr. Baker.*—What you gave him?

*Dr. Fillebrown.*—Yes.

*President Bradley.*—How frequently have you given him instructions?

*Dr. Fillebrown.*—A dozen times. (Patient: "Hardly so many, I think.") He was able to do as I told him between times; that is, to bring the tone up behind the soft palate, make the tone up in the head where it belongs, and use the lips to articulate with, particularly the upper lip. Following those two things, patients will be sure to get good articulation that can be heard readily, understood readily, and can be delivered without special effort. I gave him exercises to use that would surely carry the voice there every time.

*President Bradley.*—Do you use any diagram as to how to place the tongue?

*Dr. Fillebrown.*—No sir; the tongue will take care of itself if you give the right word to pronounce.

*Dr. Werner.*—Does he wear an artificial denture?

*Dr. Fillebrown.*—No, sir. You will notice two of the teeth are very short. These two were cut off and some crowns put on.

*President Bradley.*—Does Dr. Fillebrown wish to add anything to his paper?

*Dr. Fillebrown.*—I will say that I wish some member of the Academy who is an impartial observer would make such comments on these statements and as to what the patients have done as may appear proper to him. I prefer not to add any opinion of my own, and there is no time for discussion to bring it out.

## Figures and Tables

**Fig. 1. f1:**
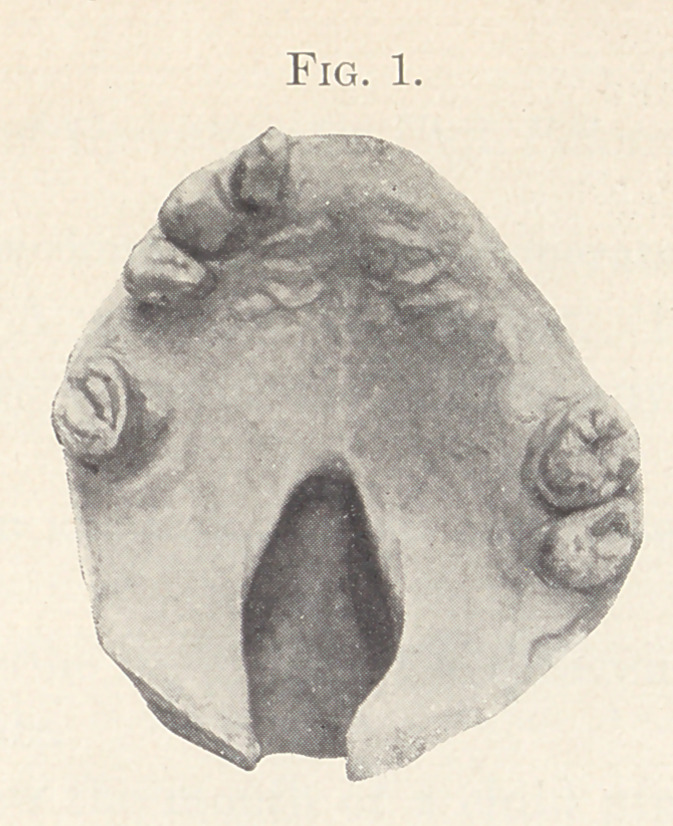


**Fig. 2. f2:**
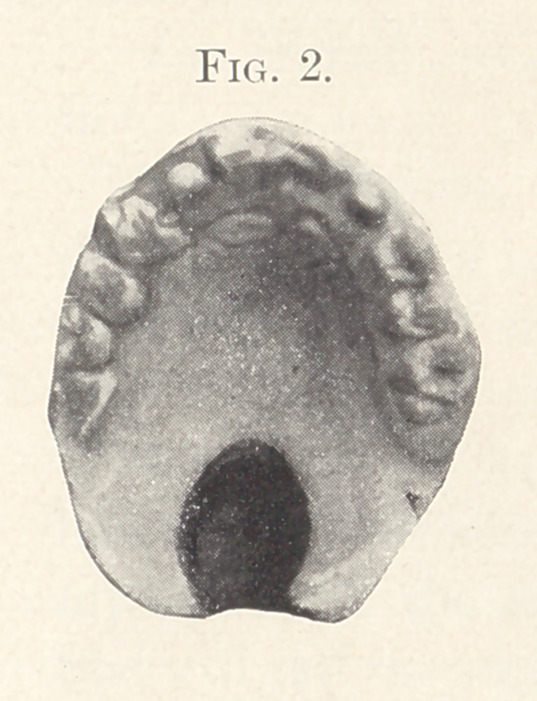


**Fig. 3. f3:**
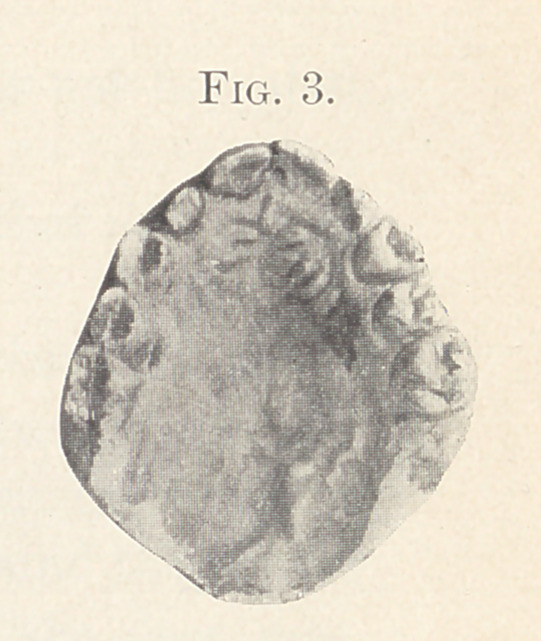


**Fig. 4. f4:**
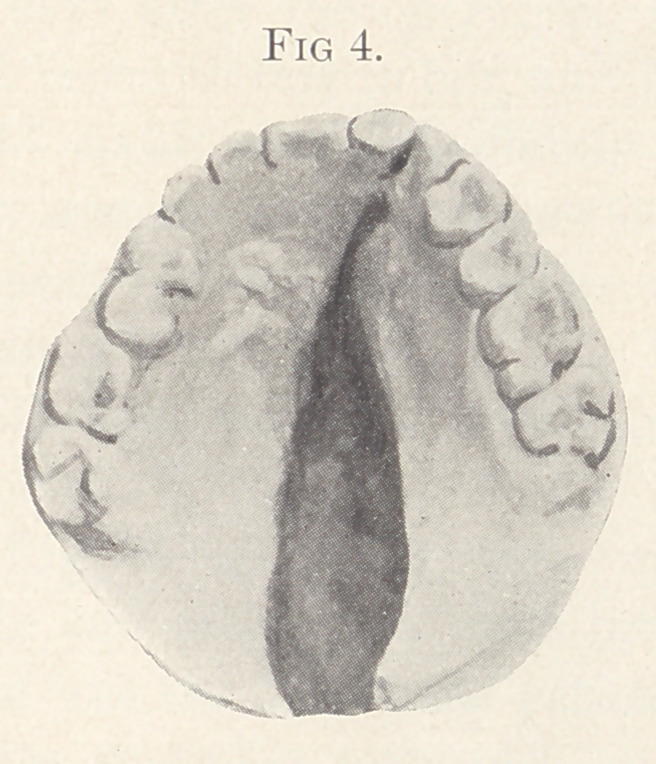


**Fig. 5. f5:**
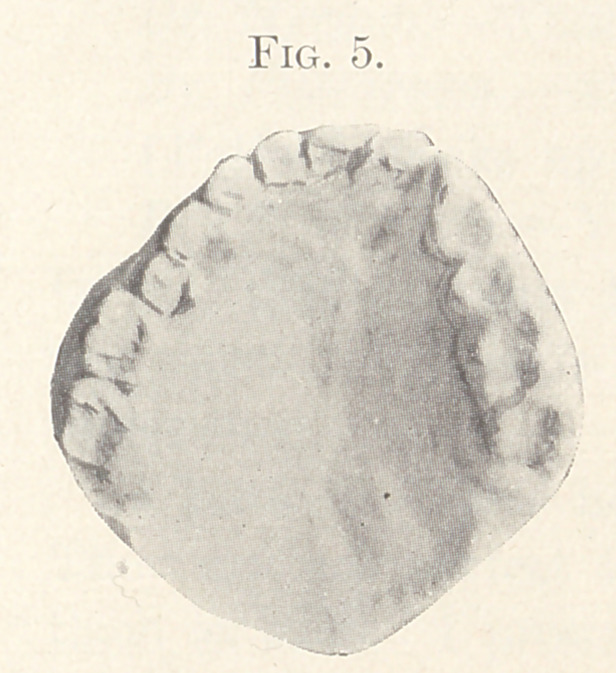


**Fig. 6. f6:**
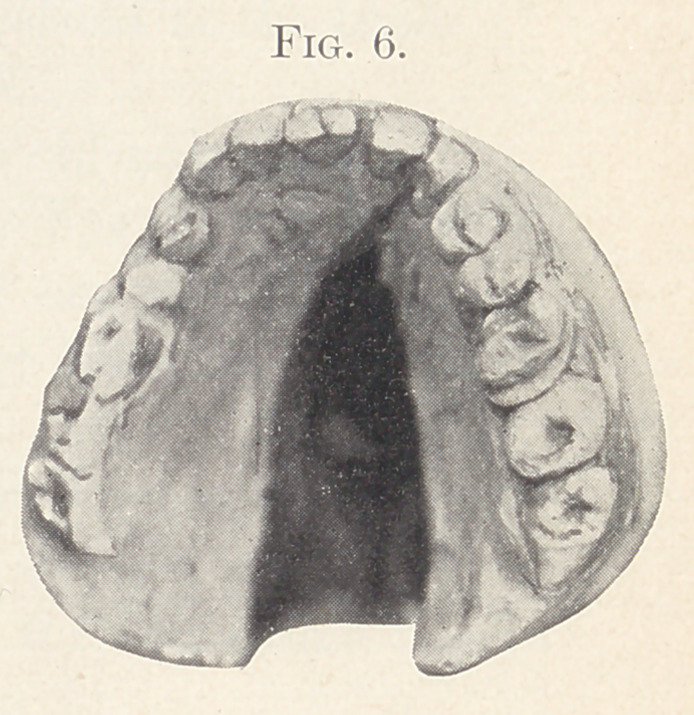


**Fig. 7. f7:**